# Hyperbaric oxygen promotes not only glioblastoma proliferation but also chemosensitization by inhibiting HIF1α/HIF2α-Sox2

**DOI:** 10.1038/s41420-021-00486-0

**Published:** 2021-05-13

**Authors:** Pan Wang, Sheng Gong, Jinyu Pan, Junwei Wang, Dewei Zou, Shuanglong Xiong, Lu Zhao, Qian Yan, Yangming Deng, Nan Wu, Bin Liao

**Affiliations:** 1 Department of Neurosurgery, Chongqing General Hospital, University of Chinese Academy of Sciences, Chongqing, 401147 China; 2 Chongqing Medical University, Chongqing, China; 3 Department of Oncology, Chongqing University Cancer Hospital, Chongqing, 400030 China

**Keywords:** Cancer microenvironment, CNS cancer, Chemotherapy, Cancer stem cells, CNS cancer

## Abstract

There exists a consensus that combining hyperbaric oxygen (HBO) and chemotherapy promotes chemotherapy sensitivity in GBM cells. However, few studies have explored the mechanism involved. HIF1α and HIF2α are the two main molecules that contribute to GBM malignant progression by inhibiting apoptosis or maintaining stemness under hypoxic conditions. Moreover, Sox2, a marker of stemness, also contributes to GBM malignant progression through stemness maintenance or cell cycle arrest. Briefly, HIF1α, HIF2α and Sox2 are highly expressed under hypoxia and contribute to GBM growth and chemoresistance. However, after exposure to HBO for GBM, whether the expression of the above factors is decreased, resulting in chemosensitization, remains unknown. Therefore, we performed a series of studies and determined that the expression of HIF1α, HIF2α and Sox2 was decreased after HBO and that HBO promoted GBM cell proliferation through cell cycle progression, albeit with a decrease in stemness, thus contributing to chemosensitization via the inhibition of HIF1α/HIF2α-Sox2.

## Introduction

As the most malignant tumour in the brain, glioblastoma (GBM) is characterized as highly aggressive and infiltrative^1^. Despite advancements in surgical interventions, patients still exhibit a poor prognosis^2^. Even with continuous improvements in chemotherapy, the survival time is extended by only approximately 2 months^2,3^. Previous studies ascribe this effect to the hypoxic microenvironment^4^ and the existence of GBM stem cells (GSCs)^5^, which are thought to be negative and vital factors to patient prognosis. Many approaches have been developed to alleviate the hypoxic environment^6^; however, the most promising way to address this problem still appears to be hyperbaric oxygen (HBO)^6,7^. Unfortunately, whether HBO alone promotes or inhibits GBM growth remains controversial. Stuhr et al.^8^ showed that tumour growth was inhibited after subcutaneously transplanted GBM cells were exposed to HBO. However, Wang et al.^7^ demonstrated that HBO promoted GBM growth. Therefore, we aimed to address this inconsistency first.

Despite the inconsistent results described above, there is a consensus that combining HBO and chemotherapy can reduce GBM growth since GBM cells become sensitive to chemotherapy^6,9,10^. However, few studies have explored the mechanism by which HBO promotes chemosensitization to GBM cells. Hypoxic-inducible factor-1α (HIF1α) and hypoxic-inducible factor-2α (HIF2α) are the two main molecules that contribute to GBM malignant progression under hypoxic conditions^5,11^. Moreover, Sox2, a marker of stemness, also contributes to GBM malignant progression through stemness maintenance or cell cycle arrest^12,13^. However, after exposure to HBO, whether the expression of the above factors is decreased, resulting in chemosensitization, remains unknown. Therefore, we performed a series of studies to determine whether the expression of HIF1α, HIF2α and Sox2 was decreased after HBO and whether HBO promoted GBM growth through cell cycle progression but with a decrease in stemness, thus resulting in chemosensitization under the inhibition of HIF1α/HIF2α-Sox2.

## Results

### HBO increases tumour volume but promotes chemosensitization

**Fig. 1:**
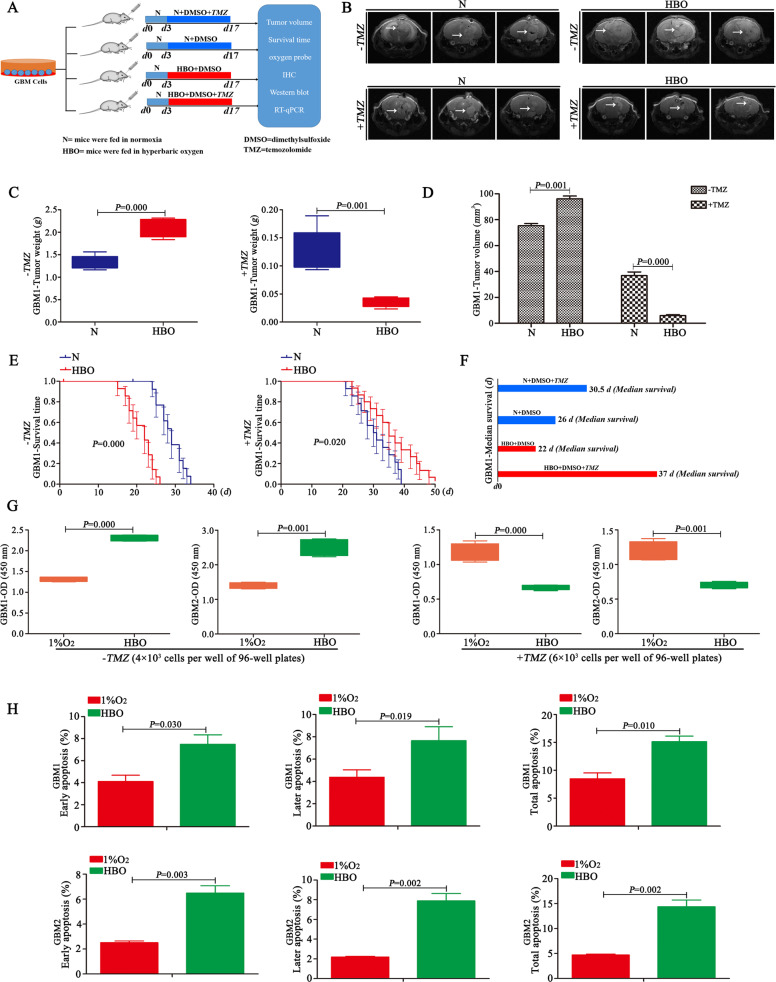
HBO increases tumour volume but promotes chemosensitization. **A** Schematic process of the in vivo study with or without HBO treatment. **B–D** MRI showed that the tumour volume was larger and the tumour weight was higher in the group under HBO conditions than in the control group without HBO treatment in the absence of TMZ. However, after treatment with the same dose of TMZ (2 mg/kg), the tumour volume decreased and the tumour weight was decreased in the group exposed to HBO compared with the control group. **E**, **F** In the absence of TMZ, the survival time became shorter after HBO exposure (median survival time of the HBO treatment group vs control group = 22 vs 26 days). However, after TMZ treatment (2 mg/kg), the group that was exposed to HBO had a much longer survival time than the control group without HBO treatment (median survival of the HBO treatment group vs control group = 37 days vs 30.5 days). **G** The cells exposed to HBO had a higher proliferation rate than the control cells without HBO treatment in the absence of TMZ, but the cells exposed to HBO had a lower growth trend than the control cells without HBO following TMZ treatment (400 μM). **H** The HBO treatment group had a higher apoptosis rate than the control group with the same TMZ treatment (400 μM). The *P* value was determined by an independent samples *t-*test, and the survival time was analysed by the log-rank test.

Magnetic resonance imaging (MRI) revealed larger tumour volumes in mice exposed to HBO than in control mice exposed to normoxia in the absence of TMZ (Fig. 1A, B, D and Supplementary Fig. 1C). In addition, tumour weights increased after HBO treatment (Fig. 1C and Supplementary Fig. 1B). Immunohistochemistry (IHC) showed that these tumour tissues highly expressed Ki67 and Bcl2 under HBO, but there were no expression of Ki67 and Bcl2 in the control group (Supplementary Fig. 1A). Survival analysis showed that the mice that were exposed to HBO had a short survival time, with a median survival time of approximately 22 days; however, survival was prolonged in control mice, with a median survival time of approximately 26 days (Fig. 1E, F and Supplementary Fig. 1D). Then, the same strategy as described above was used for the other two groups but under TMZ (2 mg/kg) treatment. MRI revealed that after 21 days, tumour volumes and weights decreased significantly in both groups after TMZ treatment; however, the tumour volumes and weights were much smaller in the group that was exposed to HBO than in the control group (Fig. 1A–D and Supplementary Fig. 1B, C). Then, the survival time of the two groups under the same TMZ treatment was analysed, and the results revealed that mice that were exposed to HBO had a longer survival time, with a median survival of approximately 37 days, than control mice, with a median survival time of only 30.5 days (Fig. 1E, F and Supplementary Fig. 1D). In addition, the in vitro study revealed a higher proliferative rate of the cells exposed to HBO than the control cells without HBO exposure in the absence of TMZ (Fig. 1G and Supplementary Fig. 1E). However, the growth trend of cells exposed to HBO was lower than that of control cells when both groups were treated with TMZ (Fig. 1G and Supplementary Fig. 1E). In addition, the HBO exposure group had a higher rate of apoptosis than the control group with the same TMZ treatment (400 μM) (Fig. 1H). Briefly, in the absence of TMZ, HBO exposure increased tumour volume and shortened the survival time; however, after TMZ was added, the group of mice exposed to HBO had a longer survival time and a smaller tumour volume.

### HBO alleviates the hypoxic environment and inhibits both HIF1α and HIF2α

**Fig. 2:**
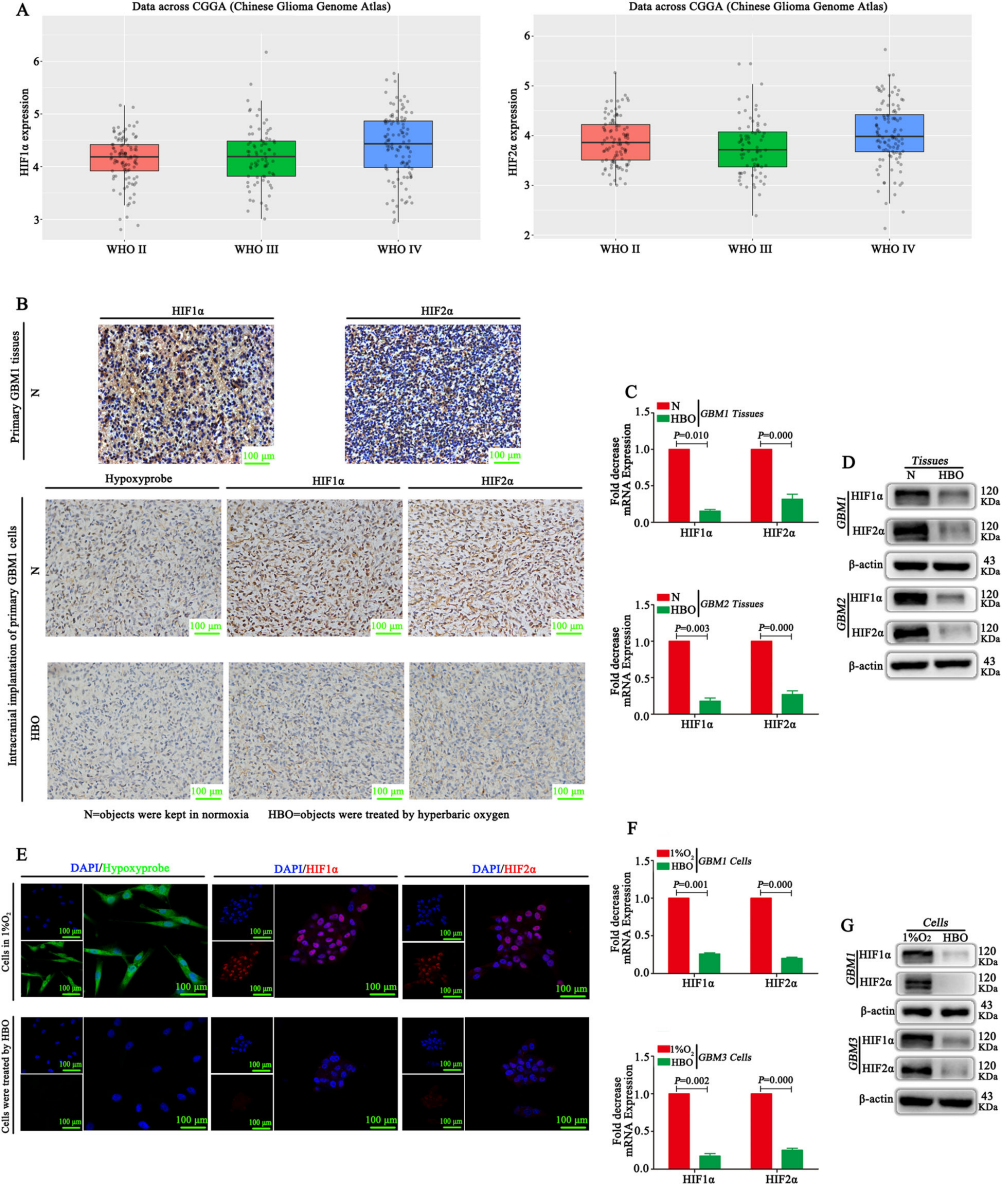
HBO alleviates the hypoxic environment and inhibits both HIF1α and HIF2α. **A** An analysis of the CGGA database showed that both HIF1α and HIF2α are highly expressed in glioma tissues. **B** HIF1α and HIF2α were highly expressed in primary GBM and tumour tissues following intracranial transplantation. Hypoxyprobe^TM^-1 was highly expressed in tumour tissues following intracranial transplantation, showing that these tissues were located in a hypoxic microenvironment. However, after HBO treatment, the hypoxic environment was alleviated based on the detection of hypoxyprobe^TM^-1, and both HIF1α and HIF2α were decreased significantly. **C**, **D** RT-qPCR and western blot showed that both HIF1α and HIF2α were decreased in tumour tissues after HBO treatment. **E** Hypoxyprobe^TM^-1, HIF1α and HIF2α were highly expressed in primary GBM cells maintained in 1% O_2_, but all of them decreased after HBO treatment according to immunofluorescence. **F**, **G** RT-qPCR and western blot showed no HIF1α or HIF2α expression in cells after HBO treatment. The *P* value was determined by an independent samples *t-*test.

CGGA database showed that both HIF1α and HIF2α were highly expressed in glioma tissues (Fig. 2A). IHC demonstrated that both HIF1α and HIF2α were highly expressed in primary GBM tissues (Fig. 2B). Tumour tissues from intracranial transplantation also showed that both HIF1α and HIF2α were highly expressed, and these tissues had high expression of hypoxyprobe^TM^-1 (Fig. 2B). However, the hypoxic environment was alleviated according to hypoxyprobe^TM^-1 results after HBO treatment, and both HIF1α and HIF2α were decreased significantly after HBO exposure according to IHC, reverse transcription quantitative PCR (RT-qPCR) and western blot results (Fig. 2B–D). Then, the vitro studies showed that these cells under hypoxic conditions highly expressed hypoxyprobe^TM^-1, HIF1α and HIF2α, whereas the cells exposed to HBO did not express hypoxyprobe^TM^-1, HIF1α and HIF2α (Fig. 2E–G).

### Inhibition of HIF1α and HIF2α increases tumour volume but promotes chemosensitization

**Fig. 3:**
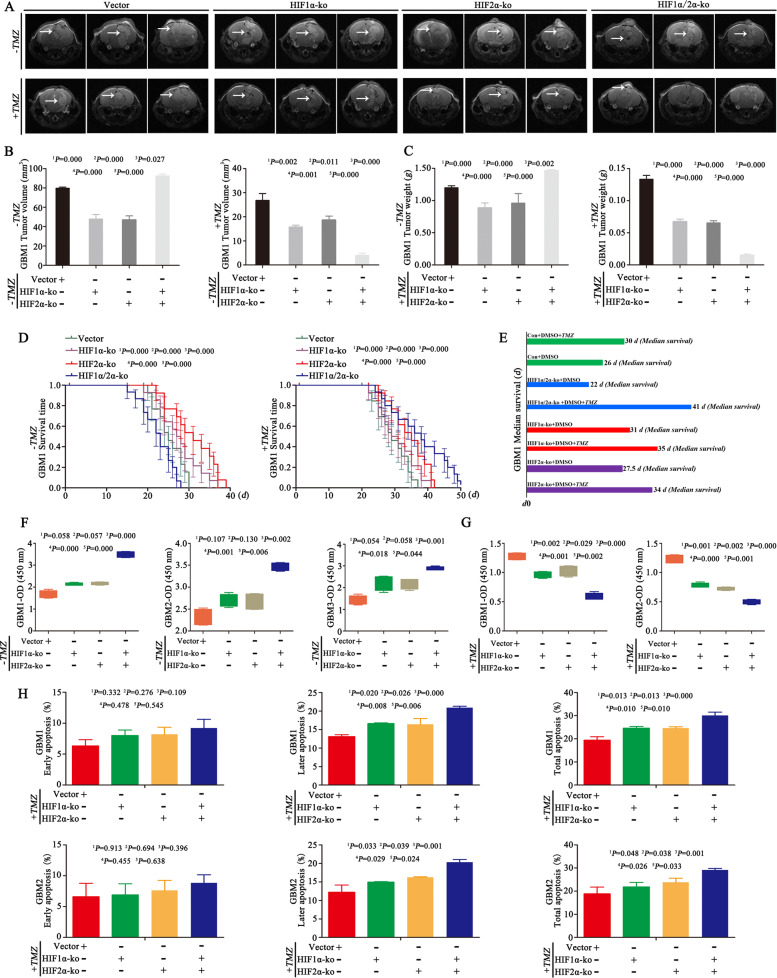
Inhibition of HIF1α and HIF2α increases tumour volume but promotes chemosensitization. **A–C** In the absence of TMZ, tumour volume and weight were decreased after HIF1α or HIF2α-KO individually compared with the empty vector control, but the dual HIF1α and HIF2α knockout group had a larger tumour volume and tumour weight than the other three groups. After TMZ treatment (2 mg/kg), the tumour volume decreased after HIF1α and HIF2α knockout individually or simultaneously, and the smallest tumour volume was observed in the group with dual HIF1α and HIF2α knockout. In addition, the group with dual HIF1α and HIF2α knockout had the lowest tumour weight among all the groups. **D**, **E** In the absence of TMZ, the survival time became longer after HIF1α or HIF2α knockout individually, but the survival time was shorter in the dual HIF1α and HIF2α knockout group than in the other three groups (median survival time of the empty vector group vs HIF1α-ko vs HIF2α-ko vs HIF1α/HIF2α-ko group = 26 vs 31 vs 27.5 vs 22 days). However, after TMZ treatment (2 mg/kg), the HIF1α or HIF2α knockout group experienced prolonged survival, and the longest survival time was observed in the group with dual HIF1α and HIF2α knockout (median survival time of the empty vector group vs HIF1α-ko vs HIF2α-ko vs HIF1α/HIF2α-ko group = 30 vs 35 vs 34 vs 41 days). **F** The above cells were cultured in 1% O_2_ for 72 h in the absence of TMZ, and there were no significant differences in cell proliferation between the HIF1α-KO, HIF2α-KO and control empty vector groups. However, after knocking out both HIF1α and HIF2α, the growth rate became the highest among all the groups. **G** The above cells were cultured in 1% O_2_ for 72 h, and TMZ (400 μM) was added to the culture medium for an additional 72 h. After HIF1α or HIF2α was knocked out individually, cell proliferation decreased. After both HIF1α and HIF2α were knocked out, the growth rate became the lowest among all the groups. **H** The above cells were cultured in 1% O_2_ for 72 h, and TMZ (400 μM) was added to the culture medium for an additional 72 h. The apoptosis rate increased after HIF1α or HIF2α knockout individually. After knocking out both HIF1α and HIF2α, the apoptosis rate became the highest among all the groups. The *P* value was determined by one-way ANOVA, and the survival time was analysed by the log-rank test.

We next wondered whether the decreased expression of HIF1α and HIF2α under HBO conditions was the reason for cell proliferation and chemosensitization. We first knocked out HIF1α and HIF2α in GBM cells and divided them into four groups: empty vector, HIF1α-KO, HIF2α-KO and dual HIF1α/HIF2α-KO (Supplementary Fig. 2A). We implanted these cells into mouse brains and found that the tumour volumes and weights of the HIF1α-ko or HIF2α-ko individually group were lower than those in the empty vector control group, but the tumour volumes of the dual HIF1α and HIF2α knockout group were significantly large, with the largest tumour weight and shortest survival time among all the groups (Fig. 3A–E). IHC showed that the tumour tissues of the HIF1α-ko or HIF2α-ko individual group had lower expression of Ki67 and Bcl2 than the control and the dual HIF1α and HIF2α knockout group (Supplementary Fig. 2B). After the intraperitoneal injection of TMZ (2 mg/kg) into the above groups, the tumour volume decreased after HIF1α or HIF2α knockout, and the smallest tumour volume was observed in the group with simultaneous HIF1α and HIF2α knockout. In addition, after TMZ treatment, the group with both HIF1α and HIF2α knockout had the lowest tumour weight and longest survival time among all groups (Fig. 3A–E). Then, the cells described above were cultured in 1% O_2_ for 72 h in the absence of TMZ, and there were no significant differences in cell proliferation between HIF1α-KO cells, HIF2α-KO cells and control empty vector cells. However, cell proliferation increased after the simultaneous knockout of HIF1α and HIF2α compared with the empty vector, HIF1α-KO and HIF2α-KO cells (Fig. 3F and Supplementary Fig. 2C). Then, we added TMZ (400 μM) to the culture medium, and the results revealed increased apoptosis after HIF1α or HIF2α knockout, and the highest apoptosis rate was observed in dual HIF1α and HIF2α knockout cells (Fig. 3H and Supplementary Fig. 2C). As a result, the proliferation rate of HIF1α-KO or HIF2α-KO cells decreased, and dual HIF1α and HIF2α knockout cells exhibited the lowest growth rate after the same TMZ treatment (400 μM) (Fig. 3G).

### Sox2 is highly expressed in GBM, and both HIF1α and HIF2α regulate Sox2 expression

**Fig. 4:**
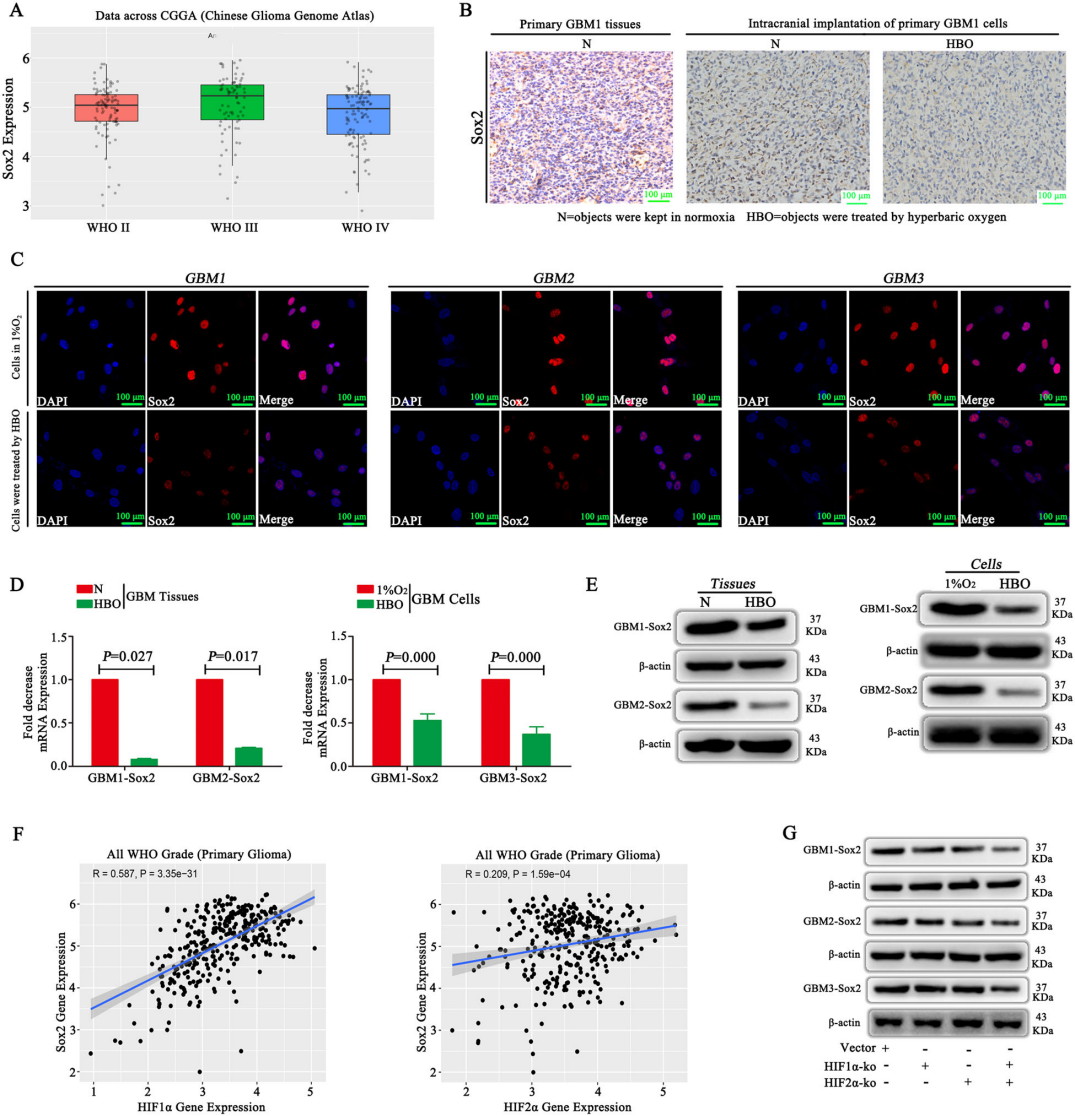
HIF1α and HIF2α regulate Sox2 expression. **A** An analysis of the CGGA database showed that Sox2 was highly expressed in GBM tissues. **B** IHC demonstrated that Sox2 was highly expressed in primary GBM and intracranial transplantation tumour tissues. However, after HBO treatment, Sox2 expression decreased significantly. **C** Sox2 was highly expressed in cells cultured in 1% O_2_, but after exposure to HBO, Sox2 levels decreased significantly. **D**, **E** RT-qPCR and western blot revealed that Sox2 expression was high in cells cultured in 1% O_2_ but decreased significantly after HBO treatment and both HIF1α and HIF2α were decreased in tumour tissues after HBO treatment. **F** Both HIF1α and HIF2α had a positive correlation with Sox2 according to analysis of the CGGA database. **G** Sox2 expression decreased after HIF1α or HIF2α knockout; however, the lowest expression was observed in the cells in which both HIF1α and HIF2α were knocked out. The *P* value was determined by an independent samples *t-*test.

CGGA database showed that Sox2 was highly expressed in GBM tissues (Fig. 4A). To compare the expression of Sox2 in the absence or presence of HBO, we collected tumour tissues from primary GBM and tumour implantation from mouse brains and observed Sox2 was expression in the group without HBO treatment; however, after HBO treatment, Sox2 expression decreased significantly (Fig. 4B). Then, the in vitro study demonstrated that Sox2 was highly expressed in the cells that were cultured in 1% O_2_, but after exposure to HBO, the Sox2 levels decreased significantly (Fig. 4C). RT-qPCR and western blot results were consistent: Sox2 expression was high in cells that were cultured in 1% O_2_ but decreased significantly after HBO treatment (Fig. 4D, E). Then, we found that both HIF1α and HIF2α had a positive correlation with Sox2 according to analysis of the CGGA database (Fig. 4F). Therefore, we cultured empty vector cells, HIF1α-KO cells, HIF2α-KO cells and HIF1α/HIF2α-KO cells under hypoxic conditions and detected Sox2 expression was lower in HIF1α-KO cells and HIF2α-KO cells than in vector control cells, and Sox2 was the lowest in the cells in which both HIF1α and HIF2α were knocked out (Fig. 4G).

### Stemness markers are highly expressed in GBM, and HBO decreases their expression

**Fig. 5:**
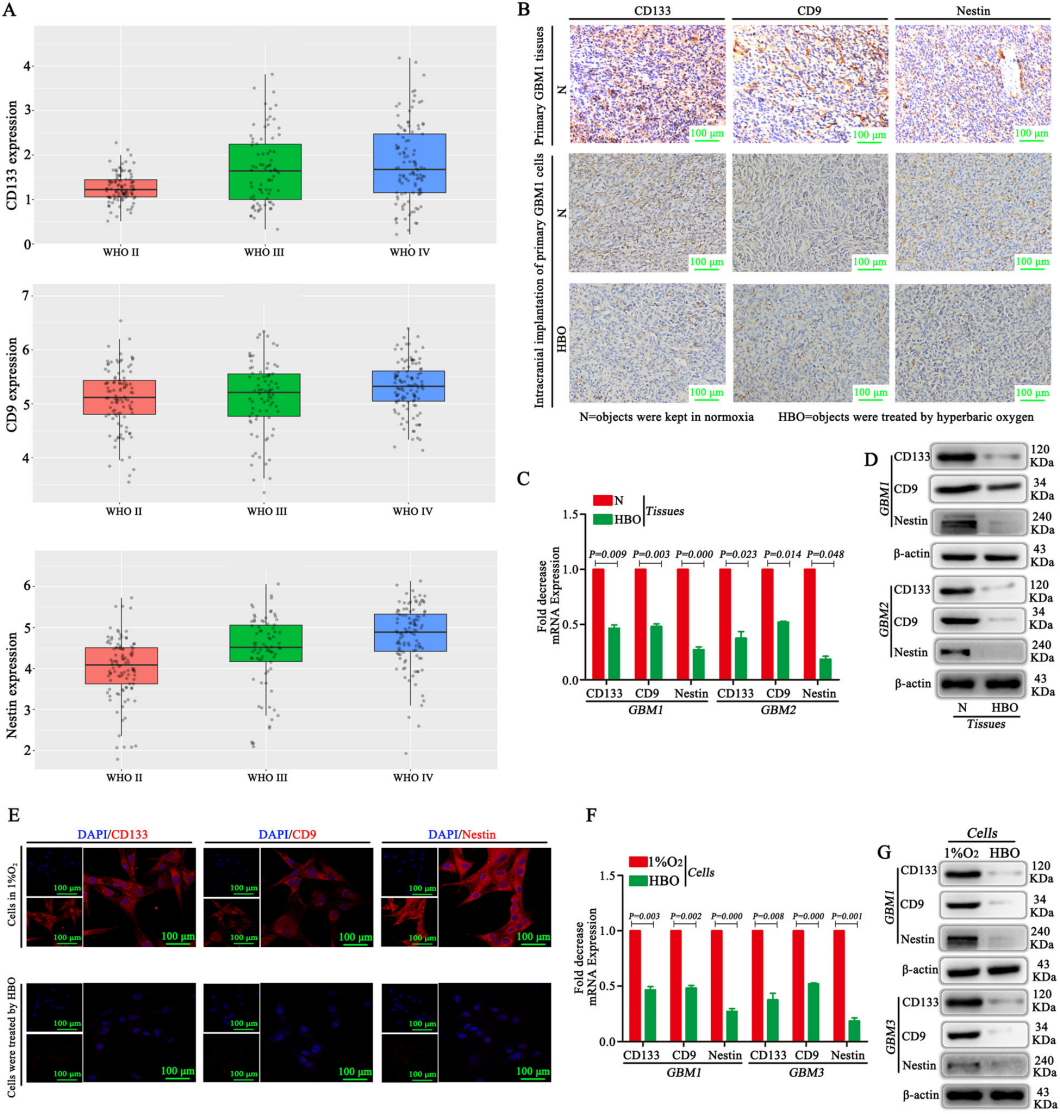
Stemness markers are highly expressed in GBM, and HBO decreases their expression. **A** An analysis of the CGGA database revealed high expression of CD133, CD9 and Nestin in GBM. **B** IHC showed that CD133, CD9 and Nestin were highly expressed in primary GBM tissues and tumour tissues from mice that underwent intracranial transplantation; however, after HBO treatment, CD133, CD9 and Nestin expression decreased significantly. **C**, **D** RT-qPCR and western blot showed that CD133, CD9 and Nestin were highly expressed in the absence of HBO in the tumour tissues from mice that underwent intracranial transplantation, but after HBO treatment, all the proteins above were significantly decreased. **E–G** The in vitro study showed that after HBO exposure, the stemness markers CD133, CD9 and Nestin decreased compared with the control without HBO treatment based on immunofluorescence, RT-qPCR and western blot results. The *P* value was determined by an independent samples *t*-test.

CGGA database and IHC showed high expression of stemness markers, such as CD133, CD9 and Nestin, in GBM (Fig. 5A, B). Then, IHC, RT-qPCR and western blotting were used to detect CD133, CD9 and Nestin in the absence or presence of HBO in the tumour tissues of mice following intracranial transplantation, and the results showed weak expression of CD133, CD9 and Nestin after HBO treatment. In contrast, if the mice were not exposed to HBO, high expression of CD133, CD9 and Nestin was observed (Fig. 5B–D). The in vitro study yielded similar results: after HBO exposure, the expression of CD133, CD9 and Nestin was lower than that in the control group without HBO treatment (Fig. 5E–G).

### HBO increases tumour volume but promotes chemosensitization due to cell cycle progression and decreases stemness by inhibiting HIF1α/HIF2α-Sox2

**Fig. 6:**
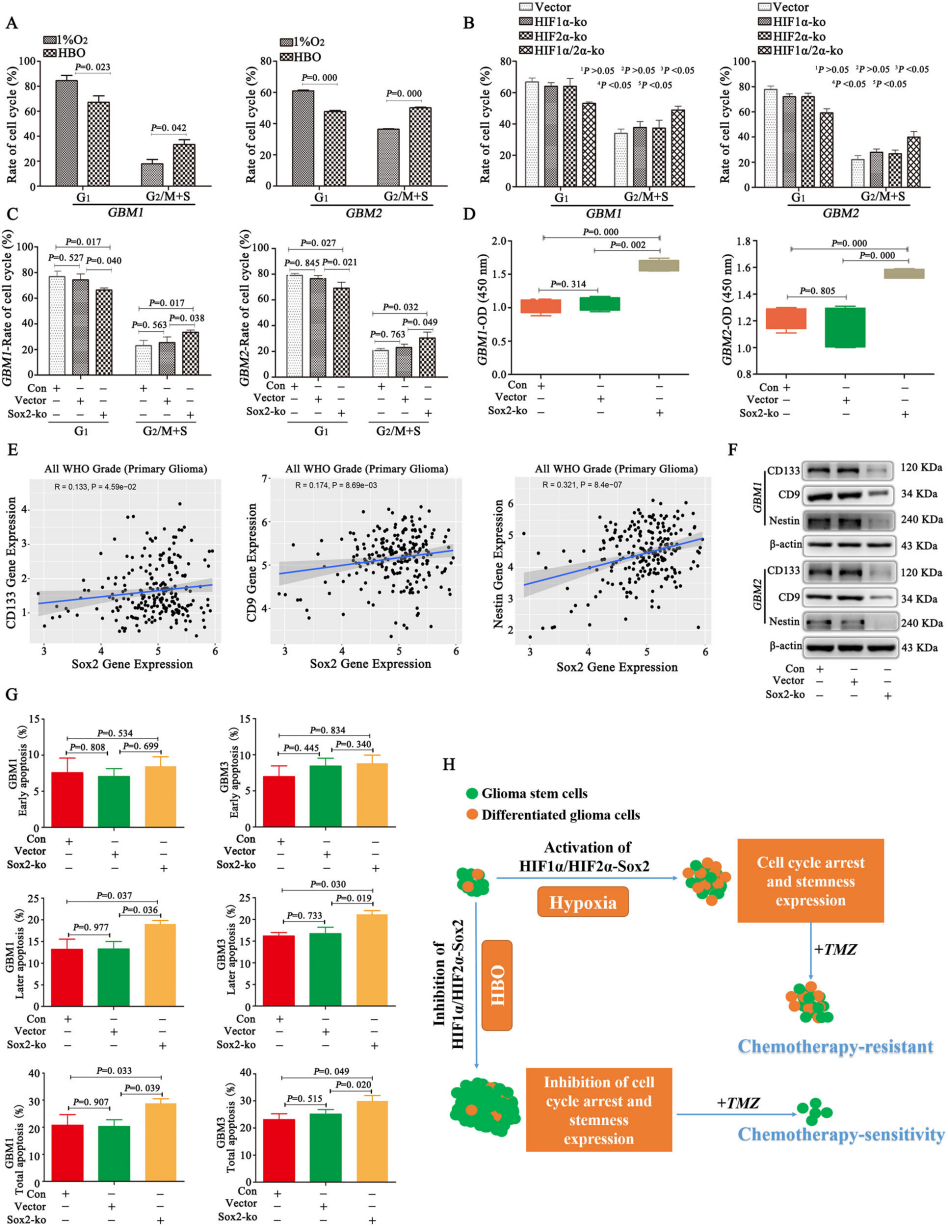
HBO increases tumour volume but promotes chemosensitization due to cell cycle progression and decreases stemness by inhibiting HIF1α/HIF2α-Sox2. **A** GBM cells after HBO exposure had a low proportion of cells in the G_1_ phase and a high proportion of cells in the G_2_/M+S phase. **B** HIF-ko cells were cultured in 1% O_2_ for 72 h, and the proportion of cells in the G_1_ phase decreased and the proportion of cells in the G_2_/M+S phase increased after dual HIF1α/HIF2α knockout. Moreover, the proportion of cells in the G_1_ phase was lower and the proportion of cells in the G_2_/M+S phase was higher in dual HIF1α/HIF2α-KO cells than in individual HIF1α- or HIF2α-KO cells. However, cell cycle changes were not significantly different between the HIF1α- or HIF2α-KO individual group and the empty vector control group. **C** There were no significant differences between the control and empty vector groups, but there was a decrease in the proportion of cells in the G_1_ phase and an increase in the proportion of cells in the G_2_/M+S phase after Sox2 knockout. **D** After Sox2 knockout in GBM cells, the proliferation rate increased (compared to control and empty vector cells). **E** A positive correlation was observed between Sox2 and CD133, CD9 and Nestin. **F** CD133, CD9 and Nestin levels decreased significantly after Sox2 knockout. **G** There were much higher late and total apoptosis rates after Sox2 knockout relative to the control. **H** GBM includes glioma stem cells and differentiated glioma cells. When these cells are cultured under hypoxia, HIF1α/HIF2α-Sox2 will become activated, thus promoting cell cycle arrest in the G_1_ phase and stemness, leading to chemotherapy resistance. However, if these cells are exposed to HBO, HIF1α/HIF2α-Sox2 will be inhibited, thus inhibiting cell cycle arrest and stemness and promoting not only cell growth but also chemotherapy sensitivity. The *P* value was determined by an independent samples *t-*test between two groups or one-way ANOVA between at least three groups.

GBM cells were cultured in 1% O_2_, and then one group of cells was maintained in 1% O_2_ while the other group was exposed to HBO. The results showed GBM cells after HBO exposure had a low proportion of cells in the G_1_ phase and a high proportion of cells in G_2_/M+S phase (Fig. 6A). We wondered whether these results were due to the decreased expression of HIF1α and HIF2α. And then we found a decrease in the proportion of cells in the G_1_ phase and an increase in the proportion of cells in the G_2_/M+S phase after HIF1α or HIF2α knockout individually; however, the change in the cell cycle in the above groups was not statistically significant. Nevertheless, the proportion of cells in the G_1_ phase was significantly lower and the proportion of cells in the G_2_/M+S phase was significantly higher in the dual HIF1α/HIF2α-ko group than in the other three groups (Fig. 6B). Because Sox2 is regulated by HIF1α and HIF2α, we investigated changes in the cell cycle after Sox2 knockout. The results showed no significant differences between the control and empty vector groups; however, the proportion of cells in the G_1_ phase decreased and the proportion of cells in the G_2_/M+S phase increased after Sox2 knockout (Fig. 6C). We investigated the influence of cell cycle changes on proliferation and found that after Sox2 knockout, the proliferation rate increased (compared to the control and empty vector) (Fig. 6D). Based on the expression of stemness markers in GBM cells and the upregulation of stemness by HIF1α and HIF2α, we wondered whether the decrease in stemness resulted from the decrease in Sox2, thus leading to chemosensitization. Therefore, we used the CGGA database and found a positive correlation between Sox2 and CD133, CD9 and Nestin (Fig. 6E). In addition, we cultured control, empty vector and Sox2-ko cells under hypoxia and found that CD133, CD9 and Nestin levels decreased significantly after Sox2 knockout (Fig. 6F). Next, to verify chemosensitization, we detected cell apoptosis, and the results showed a much higher apoptosis rate in Sox2-ko cells than in control cells (Fig. 6G).

## Discussion

Both the hypoxic environment and GSCs contribute greatly to GBM malignant progression^4,5^. Regarding the correlation between hypoxia and GSCs, previous studies have shown that the hypoxic environment maintains or induces stemness^4,14^, leading to GBM malignant progression mainly due to resistance to radiochemotherapy^15^. Therefore, hypoxia plays an important role, and it is urgent to find ways to alleviate hypoxia. Previous studies have described many ways, such as increasing the ability of red blood cells to carry oxygen or gene therapy to correct the hypoxic environment^6^. However, at present, the most effective way to alleviate hypoxia according to studies is HBO^6,9^. Unfortunately, to date, only a few studies have examined the influence of HBO alone on GBM, and there is no consensus on the issues discussed above. In 2015, Wang et al.^7^ performed a series of experiments and found that HBO promoted malignant glioma growth, thus leading to a poor prognosis. Other researchers reported the same results later^9,16^. In addition, our study demonstrated the formation of large tumours after continuous HBO treatment in vivo; these mice had a relatively shorter survival time than the control mice. However, another study from Stuhr et al.^8^ showed that the tumour volume of subcutaneously transplanted gliomas in C57Bl/6J mice was much smaller after HBO treatment than in control mice. Here, we find that subcutaneously transplanted glioma cells differ from intracranial glioma cells, and there is no doubt that the results from intracranial gliomas are better than those from subcutaneously transplanted gliomas.

Although several studies have suggested that HBO promotes GBM growth, no reports have described the detailed mechanism. According to the theory that hypoxia inhibits cell cycle progression and arrests the cell cycle in the G_1_ phase^14,17^, we focused on the influence of HBO on the cell cycle. Through a series of experiments, we found that the cell cycle progressed, with a high proportion of cells entering the G_2_/M+S phase after HBO treatment, thus leading to GBM cell proliferation and increasing tumour volume. In addition, our results revealed that the tissues or cells after HBO had a higher expression of Ki67 and Bcl2, which means that HBO promotes cell proliferation. However, the cells under HBO with TMZ treatment had a higher apoptosis rate than the control without HBO but with the same dose of TMZ, which means that HBO promotes chemosensitization. Next, we wondered why the tumours formed following HBO exposure were sensitive to chemotherapy. Hypoxia maintains the stemness of GSCs and even induces the formation of GSCs from differentiated glioma cells^5,18^. Briefly, stemness markers are highly expressed under hypoxic environments, which is why GBM cells are resistant to chemotherapy. Therefore, we detected stemness markers, including CD133, CD9 and Nestin, and the results showed that the above proteins were decreased significantly after HBO treatment. This result was consistent with that described by Zeng et al.^19^, who showed that HBO inhibited GBM stemness, with decreased sphere formation and decreased Nestin expression. Hence, according to previous studies and ours, we conclude that because of the decreased expression of stemness after HBO, GBM cells become chemosensitized to TMZ, resulting in a decrease in tumour volume, although tumour volume actually increased under HBO alone due to cell cycle progression.

The molecular mechanism is also important for understanding the sensitization of GBM cells to chemotherapy under HBO conditions. To date, only a few studies have reported the mechanism involved and demonstrated that the decrease in TNF-α, VEGF and MMP9 contributes to chemosensitization^20^. In contrast to previous studies, we focused on the molecular mechanism through changes in the hypoxic environment. HIF1α and HIF2α are the two main molecules that regulate GBM cell stemness and cell cycle arrest, promoting the resistance of GBM cells to chemotherapy^11,21^. Both HIF1α and HIF2α are steadily expressed under hypoxia, but with the increase in oxygen levels, their expression is decreased significantly^11^. However, to date, only a few reports have described the expression of HIF1α under HBO conditions in GBM cells, and no studies have reported the influence of HBO on HIF2α. Concerning the regulatory effect of HBO on HIF1α, these views remain controversial. The majority of studies have shown that HIF1α expression in GBM is decreased after HBO treatment^20,22^, but one article reported that the expression of HIF1α was increased in HBO-treated glioma tissues compared with control tissues^16^. To clearly determine the influence of HBO on the expression of HIF1α and HIF2α, we detected their expression in vivo and in vitro and found that both were decreased significantly under HBO conditions. Hence, we wondered whether the decreased expression of HIF1α and HIF2α contributed to the increase in tumour volume and chemosensitization. Through in vitro studies, we surprisingly found that after knocking out HIF1α or HIF2α individually, there was no difference in the cell cycle between control and HIF1α- or HIF2α-KO cells. However, we should care that the tumour volume was smaller for HIF1α- or HIF2α-KO individual cells in the vivo study, but there was no difference in cell proliferation compared with the control in the in vitro study, which may be due to the short detection time in vitro and the different microenvironments between the in vitro and in vivo studies. After knocking out HIF1α and HIF2α at the same time, cell cycle arrest was inhibited, and the proportion of cells in the G_2_/M+S phase increased, thus contributing to GBM cell proliferation and the increase in tumour volume. In addition, several previous studies have demonstrated that HIF1α or HIF2α knockout can inhibit the expression of GBM stemness^5,18,23^. We confirmed that knocking out HIF1α or HIF2α decreased the expression of CD133, CD9 and Nestin, especially after knocking out both HIF1α and HIF2α at the same time. According to these results, we conclude that HBO promotes not only GBM growth but also chemosensitization due to the decreased expression of both HIF1α and HIF2α by promoting cell cycle progression and inhibiting stemness.

Sox2, a transcription factor, is an indispensable element that induces the formation of induced pluripotent stem cells^24^, and their formation ability improves under hypoxic conditions^25^. Previous studies have reported that Sox2 is highly expressed in GBM cells under hypoxia and is considered a marker of stemness^12,18,26^. However, at present, its expression following HBO exposure is unclear. Therefore, we performed this research and found that Sox2 was not expressed after HBO treatment. Regarding the relationship between HIF1α and Sox2, Qiang et al.^27^ showed that HIF1α upregulates Sox2 under hypoxic conditions and promotes stemness. Regarding the HIF2α and Sox2, a few reports have demonstrated that HIF2α induces Sox2 in embryonic stem cells^28^, but the correlation between HIF2α and Sox2 in GBM is unknown. Therefore, we analysed the relationship using the CGGA database and found that both HIF1α and HIF2α exhibited a positive correlation with Sox2. Then, we cultured HIF1α- and HIF2α-knockout cells under hypoxia and found that Sox2 expression was decreased and that Sox2 expression was lowest in dual HIF1α/HIF2α-KO cells. Moreover, we knocked out Sox2 in GBM cells and cultured them in a hypoxic environment. The stem cell markers CD133, Nestin and CD9 were decreased after Sox2 inhibition, which indicates that Sox2 induces stemness in GBM cells under hypoxia. Next, regarding the cell cycle, Otsubo et al.^29^ showed that Sox2 was downregulated in gastric cancers, which led to cell cycle arrest in the G_1_ phase, thus inhibiting cell growth. In contrast, after Sox2 was overexpressed, the cell cycle progressed and promoted tumour growth. However, some studies have suggested that Sox2 promotes GBM growth as the cell cycle progresses to the S phase^13,30^. In contrast, other studies have suggested that Sox2 overexpression does not promote GBM cell proliferation, while Sox2 knockdown induces GBM cell proliferation, thereby increasing cell growth^31,32^. Concerning whether Sox2 regulates the cell cycle, we found that Sox2-ko cells had a low proportion of cells in the G_1_ phase but a high proportion of cells in the G_2_/M+S phase, promoting cell proliferation and GBM growth according to our results.

In brief, the proposed mechanism is as follows (Fig. 6H). First, HIF1α and HIF2α are highly expressed in GBM cells under hypoxia, but the expression of both HIF1α and HIF2α is inhibited after HBO treatment. Second, the cell cycle is arrested in the G_1_ phase under hypoxia, but HBO promotes cell cycle progression into the G_2_/M+S phase, thus leading to tumour growth. Third, stemness is active under hypoxia and, as a result, contributes to GBM cell chemoresistance. However, after HBO treatment, stemness is decreased, thus promoting chemosensitization. Fourth, GBM cells grow but become chemosensitive after HBO treatment due to the progression of the cell cycle but with a decrease in stemness because of the inhibition of HIF1α/HIF2α-Sox2. Fifth, not only tumour growth but also chemosensitization after HBO is ascribed to the downregulation of both HIF1α and HIF2α but not the downregulation of HIF1α or HIF2α individually. Therefore, according to our results, we conclude that HBO alone is not suitable for GBM treatment, but the combination of HBO with chemotherapy is a fine way to cure GBM and improve patient prognosis; the detailed mechanism is attributed to the inhibition of HIF1α/HIF2α-Sox2.

## Materials and methods

### Public data collection

The Chinese Glioma Genome Atlas (CGGA) database (http://www.cgga.org.cn) was used to analyse the expression of HIF1α, HIF2α, Sox2, CD133, CD9 and Nestin, and correlations among the above proteins was investigated through the dplyr, tibble and ggpolt2 packages in R.

### Cell isolation and cell culture

Primary GBM cells were isolated from tissues obtained from three different patients after surgery and anonymized, and we called them GBM1, GBM2 and GBM3; the detail information is presented in Supplementary Table 5. STR profiling was used to authenticate GBM cells and all the cells were verified none mycoplasma contamination. The tissues were minced and digested at 37 °C for 45–60 min with 0.25% trypsin (HyClone, USA) and 10 U/ml DNase I (Sigma, USA). ACK lysis buffer (Beyotime Biotechnology, China) was used to lyse red blood cells. Then, the suspension was filtered using a 100-μm cell strainer. The obtained cells were cultured in Dulbecco’s modified Eagle’s medium (DMEM)/F12 (HyClone, USA) supplemented with 10% foetal bovine serum (FBS, Gibco, USA) to maintain growth in 21% O_2_ and 5% CO_2_ at 37 °C.

### Cell treatment

Cell growth, cell cycle and protein expression using immunofluorescence and western blotting were detected after cultivation in 1% O_2_ for 72 h, and the HBO group was cultured in 1% O_2_ for 3 days but exposed to HBO for 2 h each day. mRNA expression was detected after culturing in 1% O_2_ for 12 h, and the HBO group was cultured in 1% O_2_ for 12 h but with HBO exposure 2 h before we performed mRNA detection. For cell apoptosis, we added TMZ (400 μM) to the medium of GBM cells and cultured it in 1% O_2_ for 72 h, and the HBO group treated with TMZ (400 μM) was cultured in 1% O_2_ for 3 days but exposed it to HBO for 2 h each day. GBM cells were plated into 96-well plates (4 × 10^3^ cells per well without TMZ, 6 × 10^3^ cells per well with TMZ) with DMEM/F12+10% FBS to detect cell growth using CCK-8. The cell cycle was detected using flow cytometry (FCM), protein expression was detected by immunofluorescence and western blot, and mRNA expression was detected by RT-qPCR. Detailed steps of CCK-8, FCM, immunofluorescence, western blotting and RT-qPCR are listed in the Supplementary Materials and Methods.

### Immunohistochemistry

GBM tissues obtained from patients or tumour tissues following intracranial implantation in the absence or presence of HBO were used to detect HIF1α, HIF2α, Sox2, CD133, CD9 and Nestin using IHC. The primary steps of IHC are presented in the Supplementary Materials and Methods.

### Hypoxyprobe^TM^-1 kit

Anti-human hypoxyprobe^TM^-1 (Burlington, MA, USA) was intraperitoneally injected (60 mg/kg), and the mice were fed for an additional 1 h under normoxia or HBO. Tumour tissues were fixed in 4% paraformaldehyde, embedded in paraffin, sliced and stained using IHC. For GBM cells, hypoxyprobe^TM^-1 (100 μmol/ml) was added to the medium, and the cells were cultured for an additional 1 h. Cells were fixed with 4% paraformaldehyde for 30 min and stained using immunofluorescence.

### HIF and Sox2 knockout assays

The online CRISPR design programme was used to design plasmid constructs for human HIF1α, HIF2α and Sox2 single-guide RNAs (sgRNAs). The above sgRNAs (the sequences are listed in Supplementary Table S4) were annealed and cloned into the lentiCRISPRv2 vector (Addgene, #52961, USA). The lentivirus was transfected into 293 T cells with the transducing vector, and then the vectors psPAX2 (Addgene #12260, USA) and pMD2. G were packaged (Addgene #12259, USA). Forty-eight hours after transfection, the supernatant containing particles was collected, filtered and transduced into GBM cells. Western blot analysis was performed to confirm the knockout of HIF1α, HIF2α and Sox2. The groups were as follows: control, empty vector, HIF1α-ko, HIF2α-ko, HIF1α/HIF2α-ko and Sox2-ko. Cell growth, the cell cycle and apoptosis were examined to determine the influence of the downregulation of HIF1α, HIF2α or Sox2 on GBM cells from the above groups in the absence or presence of TMZ under 1% O_2_ for 72 h. The detailed steps of the above detection are described in the Supplementary Materials and Methods.

### In vivo study

BALB/c-nu mice (male, 4–6 weeks) were used in this study. GBM cells (5 × 10^4^) were seeded into the brains of 100 nude mice, and the mice were randomly divided into four groups: normoxia+DMSO, normoxia+DMSO+TMZ, HBO+DMSO and HBO+DMSO+TMZ (*n* = 25 mice per group). TMZ (2 mg/kg) was injected into the enterocoelia every day from days 3 to 17 in the TMZ treatment groups. HBO was also administered from days 3 to 17 in the HBO treatment groups. Mice in the TMZ and HBO groups were injected with TMZ (2 mg/kg) within 30 min after HBO exposure. HBO was administered at a pressure of 2.5 ATM for a total of 90 min. Fifteen minutes of pressurization and depressurization were given so that the mice adjusted to changes in pressure. Continuous HBO treatment included a 5–10 min ramp-up to 2.5 ATM under a 100% O_2_ environment, followed by sustaining for 90 min at 2.5 ATM, followed by a 10–20 min decompression phase (these steps were performed in accordance with the in vitro study).

Tumour volume was assessed in five mice after feeding for 21 days using MRI. Five mice from each group were sacrificed on day 18, and tumour tissues were collected to analyse the weight and the expression of HIF1α, HIF2α and Sox2 using RT-qPCR, western blot and IHC. The remainder of the mice were used to analyse survival.

Control empty vector cells, HIF1α-KO cells, HIF2α-KO cells and dual HIF1α/HIF2α-KO cells (5 × 10^4^) were injected into the brains of the mice. The mice were randomly divided into eight groups, with 21 mice per group (Con+DMSO, Con+DMSO+TMZ, HIF1α-ko+DMSO, HIF1α-ko+DMSO+TMZ, HIF2α-ko+DMSO, HIF2α-ko+DMSO+TMZ, dual HIF1α/HIF2α-ko+DMSO, and dual HIF1α/HIF2α-ko+DMSO+TMZ) and fed under an environment of 21% O_2_. MRI was performed to assess tumour volume on day 21 in five mice. Five mice from each group were sacrificed on day 18, tumour tissues were collected to analyse the weight, and the remainder of the mice were used to analyse survival. TMZ (2 mg/kg) was injected into the enterocoelia every day from days 3 to 17 in the TMZ treatment groups.

### Statistical analysis

SPSS 19.0 was used for statistical analysis, and the data are presented as the mean ± standard deviation (mean ± SD). Student’s *t-*test was used to analyse the significance of differences between two groups, and one-way analysis of variance (one-way ANOVA) was utilized to analyse the significance of differences between at least three groups. The log-rank test was used for survival analysis. Correlations among HIF1α, HIF2α and Sox2 were determined according to Pearson’s analysis. *P* < 0.05 was considered statistically significant.

## Supplementary information

Supplementary_Figure_legends

Supplementary_materials_and_methods

Table S1

Table S2

Table S3

Table S4

Table S5

Sup 1

Sup 2
