# Supplementary material for: Hyperbaric oxygen promotes not only glioblastoma proliferation but also chemosensitization by inhibiting HIF1α/HIF2α-Sox2
Source: Cell Death Discov. 2021 May 13;7:103. doi: 10.1038/s41420-021-00486-0 (PMC8119469; doi:10.1038/s41420-021-00486-0)
Supplement: Supplementary file 1 — Supplementary_Figure_legends [file 41420_2021_486_MOESM1_ESM.docx]

**Supplementary Figure 1 HBO increases tumour volume but promotes chemosensitization. A** IHC showed that these tumour tissues highly expressed Ki67 and Bcl2 under HBO, but there were no expression of Ki67 and Bcl2 in control group **B-C** The tumour weights were higher and the tumour volumes were larger in the HBO treatment group than in the control group in the absence of TMZ. However, after the same dose of TMZ (2 mg/kg), the tumour volume and tumour weight were lower in the group with HBO exposure than in the control group. **D** Without TMZ treatment, the survival time became shorter after HBO treatment (median survival time of the HBO treatment group *vs* control group= 20.5 days *vs* 29 days). However, after TMZ treatment (2 mg/kg), the group that was exposed to HBO had a much longer survival time than the control group without HBO treatment (median survival time of the HBO treatment group *vs* control group= 43 days *vs* 34 days). **E** The cells treated with HBO had a higher proliferation rate than the control cells without HBO treatment in the absence of TMZ, and the cells treated with HBO had a lower growth trend than the control cells without HBO treatment in the presence of TMZ (400 μM).

**Supplementary Figure 2 A** Western blot showed successful HIF1α knockout in HIF1α-KO cells and HIF1α/HIF2α-KO cells; Western blot analysis showed successful HIF2α knockout in HIF2α-KO cells and HIF1α/HIF2α-KO cells. **B** IHC showed that the tumour tissues of the HIF1α-ko or HIF2α-ko individual group had lower expression of Ki67 and Bcl2 than the control and the dual HIF1α and HIF2α knockout group. **C** *P* value among empty vector, HIF1α-KO, HIF2α-KO and HIF1α/HIF2α-KO cells for Figure 3 and Figure 6B.

**Supplementary Table 1** Primary antibodies used in immunofluorescence or IHC

**Supplementary Table 2** Primary antibodies used in western blotting

**Supplementary Table 3** The sequences of primers used for RT-qPCR detection

**Supplementary Table 4** The sequences of sgRNA for knockout of HIF1α, HIF2α and Sox2

**Supplementary Table 5** The detail information of GBM patients
