# Supplementary material for: Hyperbaric oxygen promotes not only glioblastoma proliferation but also chemosensitization by inhibiting HIF1α/HIF2α-Sox2
Source: Cell Death Discov. 2021 May 13;7:103. doi: 10.1038/s41420-021-00486-0 (PMC8119469; doi:10.1038/s41420-021-00486-0)
Supplement: Supplementary file 2 — Supplementary_materials_and_methods [file 41420_2021_486_MOESM2_ESM.docx]

**Materials and methods**

**Immunohistochemistry (IHC)**

HIF1α, HIF2α, Sox2, CD133, CD9 and Nestin were detected in GBM tissues obtained from patients or following intracranial implantation using IHC. The primary steps were as follows: formalin fixation, paraffin embedding, dewaxing in xylene, rinsing in graded ethanol and rehydrating in double-distilled water. Next, antigen retrieval was performed, and slides were pretreated with sodium citrate buffer for 15 min at 95°C. Then, the slides were washed with PBS for 3 min and immunostained using primary antibodies against HIF1α, HIF2α, Sox2, CD133, CD9 and Nestin (related information regarding primary antibodies is presented in Supplementary Table S1) at 4°C overnight. The slides were washed with PBS again, and the tumour sections were covered with HRP-conjugated anti-mouse/rabbit antibodies for 2 h. Samples were covered with DAB chromogen solution and incubated for ~1 min, and images were obtained at the end of the procedure.

**Immunofluorescence**

HIF1α, HIF2α, Sox2, CD133, CD9 and Nestin were detected in GBM cells following exposure to hypoxia or HBO through immunofluorescence. The cells were fixed in 4% paraformaldehyde at 4°C (30 min), washed twice with PBS containing 0.5% Triton X-100 (Sigma, USA) and blocked with 10% normal serum. The cells were then incubated at 4°C for 24 h with primary antibodies against HIF1α, HIF2α, Sox2, CD133, CD9 and Nestin (related information regarding primary antibodies is presented in Supplementary Table S1). Cells were washed with PBS again at least three times for 5 min and incubated at 37°C for an additional 2 h with fluorophore-labelled secondary antibodies (CST, USA). A laser scanning confocal microscope was used for imaging (LSM780, ZEISS, Germany).

**Western blot**

Total protein was prepared using prechilled RIPA buffer (Beyotime Biotechnology, China), subjected to SDS-PAGE, transferred to nitrocellulose membranes, blocked with 5% nonfat milk, and incubated with primary antibodies against HIF1α, HIF2α, Sox2, CD133, CD9 and Nestin (related information regarding primary antibodies is presented in Supplementary Table S2) at 4°C overnight. HRP-labelled secondary antibodies (Beyotime Biotechnology, China) were added to the medium and incubated at 37°C for an additional 1 h. Enhanced chemiluminescence was conducted for visualization.

**Real-time quantitative polymerase chain reaction (RT-qPCR)**

Total RNA was prepared using TRIzol (Invitrogen, USA), melted at 94°C for 5 min, denatured at 94°C for 30 s, annealed at 57°C for 30 s and extended at 72°C for 30 s for a total of 40 cycles. The primer sequences are presented in Supplementary Table S3.

**Flow cytometry (FCM) analysis**

Cells were digested using 0.25% trypsin and prepared as a single-cell suspension in PBS. Cells were suspended at a density of 5 × 10^5^ cells/ml and fixed with 75% ethanol at 4°C for an additional 24 h. Cells were centrifuged and washed with PBS. The cells were centrifuged again and suspended in 1 ml propidium iodide (PI) staining solution and incubated at 37°C for 30 min. GBM cells were exposed to TMZ (400 μM) in the absence or presence of HBO, and apoptosis was detected. Cells were digested with 0.25% trypsin and prepared as a single-cell suspension in PBS at 4°C. Cells were suspended at a density of 1×10^6^ cells/ml, and 100 μl of the suspension was added to an Eppendorf tube. Next, 0.05% trisodium citrate-dihydrate (195 μl) and 5 μl annexin V-FITC were added to centrifuged cells in suspension, and the mixture was placed and incubated for 15 min in the dark at room temperature (RT). Trisodium citrate-dihydrate (0.05%, 190 μl) and PI (10 μl) were added to the centrifuged cell suspension and incubated for 10 min in the dark at RT. FACS (BD Accuri C6, Germany) was used to examine the cell cycle and apoptosis.

**CCK-8 assay**

CCK-8 assays were performed to examine cell proliferation in the absence or presence of TMZ (400 μM) with or without HBO. The suspension was mixed with 10 µl of CCK-8 reagent and 90 µl of DMEM/F12+10%, and the suspension was added to each well. Cells were then cultured for an additional 2 h under 1% O_2_. An ELISA reader (Varioskan Flash, Thermo Scientific, USA) was used to measure the absorbance at 450 nm.
