# Supplementary material for: Hyperbaric oxygen promotes not only glioblastoma proliferation but also chemosensitization by inhibiting HIF1α/HIF2α-Sox2
Source: Cell Death Discov. 2021 May 13;7:103. doi: 10.1038/s41420-021-00486-0 (PMC8119469; doi:10.1038/s41420-021-00486-0)
Supplement: Supplementary file 3 — Table S1 [file 41420_2021_486_MOESM3_ESM.docx]

Table S1 Primary antibodies used in immunofluorescence or IHC

| Antigens | Manufacturer | Catalogue numbers | Application |
| --- | --- | --- | --- |
| HIF1A | abcam | ab179483 | 1:100 |
| HIF2A | abcam | ab207607 | 1:100 |
| CD133 | NOVUS | NB120-16518 | 1:100 |
| NESTIN | CST | 33475S | 1:100 |
| CD9 | abcam | ab92726 | 1:150 |
| Sox2 | R&D Systems | MAB2018 | 1:100 |
