# Supplementary material for: Hyperbaric oxygen promotes not only glioblastoma proliferation but also chemosensitization by inhibiting HIF1α/HIF2α-Sox2
Source: Cell Death Discov. 2021 May 13;7:103. doi: 10.1038/s41420-021-00486-0 (PMC8119469; doi:10.1038/s41420-021-00486-0)
Supplement: Supplementary file 5 — Table S3 [file 41420_2021_486_MOESM5_ESM.docx]

Table S3 The sequences of primers used for RT-qPCR detection

| HIF1A | Forward(5'-3') | ACTGCACAGGCCACATTCACG |
| --- | --- | --- |
|  | Reverse(5'-3') | AATCAGCACCAAGCAGGTCATAGG |
| HIF2A | Forward(5'-3') | GCGACCATGAGGAGATTCGTGAG |
|  | Reverse(5'-3') | CAGGTGGCTGACTTGAGGTTGAC |
| CD133 | Forward(5'-3') | GCCCCCAGGAAATTTGAGGAAC |
|  | Reverse(5'-3') | GCTTTGGTATAGAGTGCTCAGTGATTG |
| CD9 | Forward(5'-3') | CCCGGTCGTGCGTGCCTCTTGTC |
|  | Reverse(5'-3') | GCCGGCGAGCCAGAAGATGAAGTTA |
| Nestin | Forward(5'-3') | GCCCCTGGTGGAAGATGATG |
|  | Reverse(5'-3') | GCCCTGAACCCTCTTTGCCTC |
| Sox2 | Forward(5'-3') | GGAGGGGTGCAAAAGAGGAGAG |
|  | Reverse(5'-3') | TCCCCCAAAAAGAAGTCCAGG |
| β-actin | Forward(5'-3') | ACCCGCCGCCAGCTCACC |
|  | Reverse(5'-3') | GGGGGGCACGAAGGCTCATC |
