# Supplementary material for: Hyperbaric oxygen promotes not only glioblastoma proliferation but also chemosensitization by inhibiting HIF1α/HIF2α-Sox2
Source: Cell Death Discov. 2021 May 13;7:103. doi: 10.1038/s41420-021-00486-0 (PMC8119469; doi:10.1038/s41420-021-00486-0)
Supplement: Supplementary file 6 — Table S4 [file 41420_2021_486_MOESM6_ESM.docx]

Table S4 The sequences of sgRNA for knockout of HIF1α, HIF2α and Sox2

| Target | Oligonucleotide sequence(5'-3') |
| --- | --- |
| HIF1A | GAACTCACATTATGTGGAAG |
| HIF2A | CTTGGAGGGTTTCATTGCCG |
| SOX2 | AAAGTTTCCACTCGGCGCCC |
