# Supplementary material for: Hyperbaric oxygen promotes not only glioblastoma proliferation but also chemosensitization by inhibiting HIF1α/HIF2α-Sox2
Source: Cell Death Discov. 2021 May 13;7:103. doi: 10.1038/s41420-021-00486-0 (PMC8119469; doi:10.1038/s41420-021-00486-0)
Supplement: Supplementary file 7 — Table S5 [file 41420_2021_486_MOESM7_ESM.docx]

Table S5 The detailed pathological information of the GBM patients assessed in this study

Tissue

Content

|  | | GBM-1 | GBM-2 | GBM-3 |
| --- | --- | --- | --- | --- |
| Sex | | Female | Male | Female |
| Age | | 31 years old | 14 years old | 71 years old |
| Primary or recurrent tumor | | Primary tumor | Primary tumor | Primary tumor |
| Pathology | GFAP | + | + | + |
|  | Olig-2 | + | + | + |
|  | Ki67 | 15% | 30% | 30% |
|  | P53 | \ | \ | \ |
|  | MGMT Methylation | - | \ | \ |
|  | IDH1-132 | Mutant type | Wild type | Wild type |
|  | IDH1-127 | \ | Wild type | Wild type |
|  | IDH2-172 | \ | Wild type | Wild type |
|  | 1p/19q Codeletion | + | - | - |
| Integrated  diagnosis | | WHO III,  IDH-1 Mutant type | WHO IV,  IDH-1/2 Wild type | WHO IV,  IDH-1/2 Wild type |
