# Supplementary figures and images for: Hyperbaric oxygen promotes not only glioblastoma proliferation but also chemosensitization by inhibiting HIF1α/HIF2α-Sox2
Source: Cell Death Discov. 2021 May 13;7:103. doi: 10.1038/s41420-021-00486-0 (PMC8119469; doi:10.1038/s41420-021-00486-0)

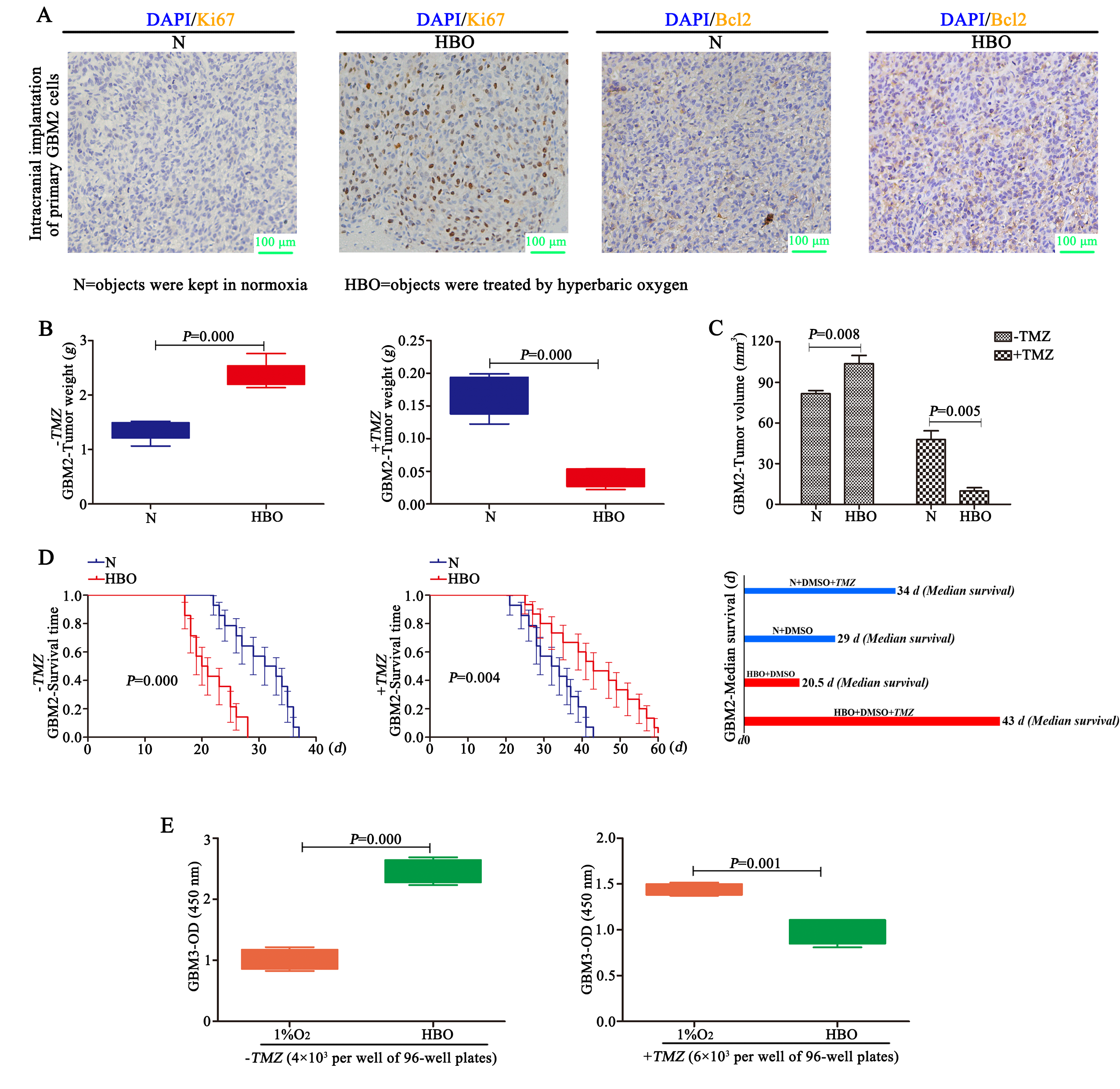

Supplement: Supplementary file 8 — Sup 1 [file 41420_2021_486_MOESM8_ESM.tif]

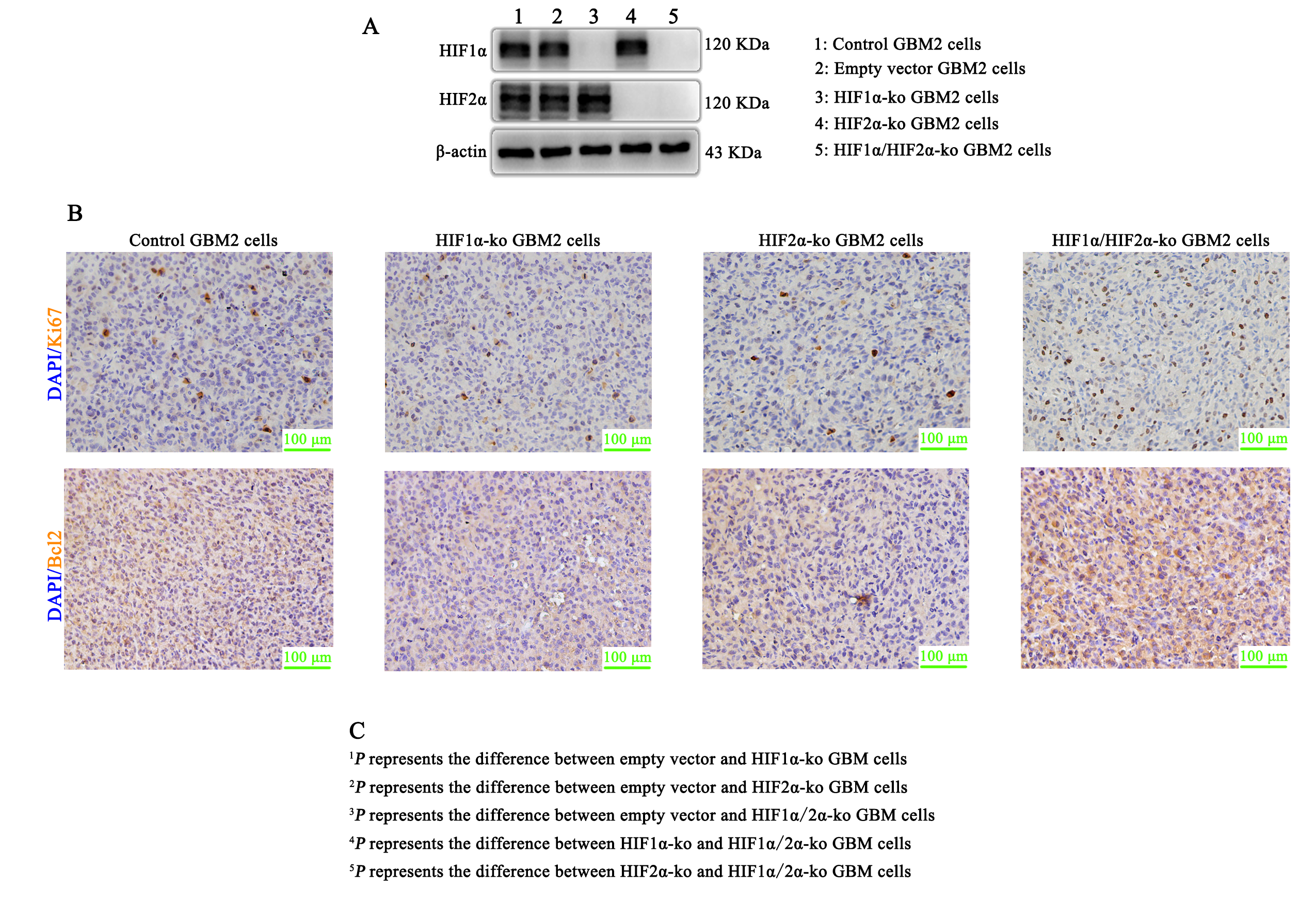

Supplement: Supplementary file 9 — Sup 2 [file 41420_2021_486_MOESM9_ESM.tif]
